# Rapamycin Enhances Mitophagy and Attenuates Apoptosis After Spinal Ischemia-Reperfusion Injury

**DOI:** 10.3389/fnins.2018.00865

**Published:** 2018-12-03

**Authors:** Qiang Li, Shane Gao, Zhanrong Kang, Meiyan Zhang, Xin Zhao, Yu Zhai, Jianming Huang, Guo-Yuan Yang, Wanju Sun, Jian Wang

**Affiliations:** ^1^Department of Neurology, Shanghai Ninth People’s Hospital, Shanghai Jiao Tong University School of Medicine, Shanghai, China; ^2^East Hospital, Tongji University School of Medicine, Shanghai, China; ^3^Department of Orthopedics, Shanghai Pudong Hospital, Fudan University Pudong Medical Center, Shanghai, China; ^4^Department of Orthopedics, Shanghai Ninth People’s Hospital, Shanghai Jiao Tong University School of Medicine, Shanghai, China; ^5^Neuroscience and Neuroengineering Research Center, Med-X Research Institute and School of Biomedical Engineering, Shanghai Jiao Tong University, Shanghai, China; ^6^Department of Orthopedics, Shanghai Pudong New Area People’s Hospital, Shanghai University of Medicine & Health Science, Shanghai, China

**Keywords:** apoptosis, mitophagy, ischemia-reperfusion injury, rapamycin, spinal cord

## Abstract

The spinal cord is extremely vulnerable to ischemia-reperfusion (I/R) injury, and the mitochondrion is the most crucial interventional target. Rapamycin can promote autophagy and exert neuroprotective effects in several diseases of the central nervous system. However, the impact of rapamycin via modulating mitophagy and apoptosis after spinal cord ischemia-reperfusion injury remains unclear. This study was undertaken to investigate the potential role of rapamycin in modulating mitophagy and mitochondria-dependent apoptosis using the spinal cord ischemia-reperfusion injury (SCIRI) mouse model. We found that rapamycin significantly (*p* < 0.05) enhanced mitophagy by increasing the translocation of p62 and Parkin to the damaged mitochondria in the mouse spinal cord injury model. At the same time, rapamycin significantly (*p* < 0.05) decreased mitochondrial apoptosis related protein (Apaf-1, Caspase-3, Caspase-9) expression by inhibiting Bax translocation to the mitochondria and the release of the cytochrome c from the mitochondria. After 24 h following SCIRI, rapamycin treatment reduced the TUNEL^+^ cells in the spinal cord ischemic tissue and improved the locomotor function in these mice. Our results therefore demonstrate that rapamycin can improve the locomotor function by promoting mitophagy and attenuating SCIRI -induced apoptosis, indicating its potential therapeutic application in a spinal cord injury.

## Introduction

Spinal cord ischemia-reperfusion injury is a severe clinical complication in surgical interventions of aortic diseases (Turkoz et al., 2007; Cheng et al., 2009; Dublin et al., 2010). Neurons in the spinal cord are vulnerable to ischemic injury because of a high demand for energy. Mitochondria have been proposed to be the principal subcellular target of a ischemia-reperfusion injury. They are essential not only for generating ATP, but also involved in pathophysiological processes of cell death (Nakai et al., 1997). Functional alterations in the mitochondria resulted in an ATP level reduction, Ca^2+^ homeostasis damage, ROS stress injury and cell apoptosis(Anne Stetler et al., 2013). Therefore, mitochondria have an enormous potential causing cause severe cell damage and play an important role in the pathophysiological process of SCIRI. It is believed that pharmacological agent targeting on mitochondria is one of the most promising approaches for SCIRI therapy.

Mitophagy, the selective clearance of dysfunctional mitochondria by autophagy, is extremely important for controlling the quality and quantity of mitochondria and promoting cell survival (Yuan et al., 2015). Using the rat middle cerebral occlusion (MCAO) mode, electron microscopy has shown that the damaged mitochondria is surrounded by autophagosomes in the ischemic penumbra, suggesting that the mitochondria are degraded by autophagy. Studies also demonstrated that mitophagy activation via the Parkin translocation mitochondria pathway can inhibit cerebral ischemia-reperfusion injury (Zhang et al., 2013). Mitophagy has been implicated in the pathophysiological process of ischemia reperfusion injury and hemorrhagic stroke injury (Zhang et al., 2013; Li et al., 2017, 2018). Therefore, mitophagy may play a pivotal role in neuronal survival during SCIRI.

Rapamycin is widely used as an inducer of autophagy, acting through its inhibitory effect on a mTOR (Mizushima et al., 2008). Increasing studies reported that rapamycin reduced neuronal death by activating the autophagy process in the injured spinal cord (Sekiguchi et al., 2012; Song et al., 2015). There is cross-talk between the autophagy and apoptosis through the mitochondria. In our previous study, we found that rapamycin reduced mitochondrial dysfunction through activating mitophagy in transient MCAO model (Li et al., 2014). However, the potential neuroprotective effect of rapamycin via activating mitophagy following SCIRI, need be clarified. Furthermore, the mechanism of modulating the dynamic balance between mitophagy and apoptosis by rapamycin after SCIRI, remains to be elucidated. Thus, the present study was undertaken to investigate the neuroprotective role of rapamycin via the activation of mitophagy and the inhibition of mitochondria-dependent apoptosis in the SCIRI mice.

## Materials and Methods

### Animals and Experimental Protocol

**FIGURE 1:**
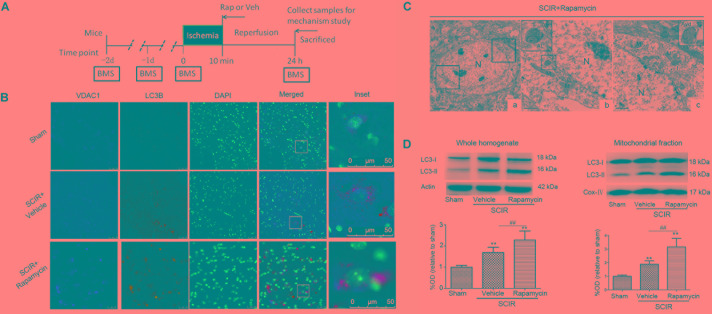
Rapamycin treatment enhanced mitophagy after spinal cord ischemia-reperfusion injury. **(A)**. Schematic diagram of the experimental design. Experimental groups were treated with vehicle and rapamycin (intraperitoneal injection, 1mg/kg). Mice received treatmetn at reperfusion onset and were sacrificed at 24 h after SCIRI. **(B)** Immunohistochemical localization of VDAC1 (mitochondrial marker) and LC3B (autophagy marker) co-staining in spinal cord at 24 h after SCIRI. VDAC1 (red), LC3B (green). The inset images represent higher magnification of the boxed area in the corresponding merged images. **(C)**. Electron micrographs demonstrating neuron mitophagy structure in rapamycin-treated SCIRI mice. Autophagic vacuoles, autolysosomes (AL), typical mitophagy structure and swollen mitochondria (black arrow)were observed in neurons. High magnifications in **(b)** revealed a typical autolysosomes structure. High magnifications in **(c)** revealed a typical mitophagy structure, that is a degradative autophagic vacuole (Avd) including a partially degraded mitochondrial. **(b)** is the high magnification of lower-left box in the figure **C-a** and **(C)** is the high magnification of upper-right box in the figure **C-a**. M-mitochondrial, N-nuclear, V-vacuole. scale bar = 0.5 μm or 2 μm. **(C)** Representative western blots and quantitative graphs demonstrate the expression of LC3-I /LC3-II in the whole homogenates at 24 h after SCIRI. **(D)** Representative western blots and quantitative graphs demonstrate the expression of LC3-I and LC3-II in the mitochondrial fractions at 24 h after SCIRI. Data were shown as mean ± SD, *n* = 6. ^∗^*p* < 0.05, ^∗∗^*p* < 0.01 vs. Sham; ^#^*p* < 0.05, ^##^*p* < 0.01.

The experimental protocol was approved by the Ethical Committee of the Experimental Animal Center affiliated with the Tongji University School of Medicine and in accordance with the National Institute of Health Guide for the Care and Use of Laboratory Animals. Male C57BL/6J mice (SLAC Inc., Shanghai, China) aged between 10 and 16 weeks were used for all experiments. A total of 114 mice were randomly assigned to three groups, a schematic diagram of the experimental design is shown in Figure 1A. The sham group (n = 18) received the same surgical procedures, but no impact injury was sustained. The rapamycin (Sigma-Aldrich, St. Louis, MO, United States) treatment group (*n* = 48) received an aortic arch cross-clamping following intraperitoneal rapamycin at the onset of reperfusion (1 mg/kg, 0.5 ml aqueous solution) (Sekiguchi et al., 2012). The vehicle treatment group (*n* = 48) received an aortic arch cross-clamping, following an intraperitoneal equivalent volume of an aqueous solution.

### Spinal Cord Ischemia-Reperfusion Injury (SCIRI)

Mice were anesthetized using 2% isoflurane and placed in the supine position. During the surgery period, the core body temperature was maintained at 37.0 ± 0.5°C using a rectal temperature probe and an automatic temperature-adjusting pad (RWD Life Science, Shenzhen, China). The aortic arch was exposed using a cervicothoracic approach as previously described (Lang-Lazdunski et al., 2000; Bell et al., 2013). A clip was placed on the aortic arch distal to the left common carotid artery and the subclavian artery for 10 min. A laser Doppler blood flow monitor (Moor Instruments, Devon, United Kingdom) was placed over the left femoral artery. Successful occlusion was defined as ≥ 90% reduction in distal flow.

### Neurological Assessment

To evaluate the functional consequences of SCIRI, a locomotor rating test using the BMS was performed (Basso et al., 2006). The BMS ranges from a score of 0 for complete paraplegia to a score of 9 for normal function. An investigator blinded to the treatment group, carried out the test in the open field and the BMS scores were measured at 0, 1, 2 days pre-SCIRI and at 24 h post-SCIRI.

### Mitochondria Isolation

Mice were anesthetized with sodium pentobarbital (50 mg/kg, intraperitoneal) and perfused transcardially with saline, 24 h after the SCIRI. The spinal cord tissue (T8-L4) was removed and used to isolate the mitochondria with an animal tissue active mitochondrial extraction kit (Genmed Scientifics Inc., Shanghai, China) as previosuly described (Li et al., 2014). The spinal cord tissue was briefly homogenized with a glass homogenizer (15 20 strokes) and centrifuged at 1500 *g* for 10 min at 4°C. The supernatant was removed and centrifuged at 10,000 *g* for 10 min. Next, the supernatant was separated via a cytosolic fraction, and the pelleted materials were washed three times and suspended in 10 mM Tris-HCl, pH 7.4, containing 10 mM KCl, 0.25 M sucrose and 5 mM MgCl_2_. The protein concentration was measured using a Pierce BCA kit (Pierce, Rockford, IL, United States). The isolated mitochondrial and cytosolic fractions were collected for a western blot assay.

### Western Blot Analysis

Spinal cord tissues were collected 24 h after the SCIRI, and the whole homogenate, isolated mitochondrial and cytosolic fractions were used for Western blotting. Equal amounts of protein per lane (30 μg) were briefly subjected to electrophoresis on a 4 12% SDS–PAGE gel. Proteins were electrotransferred onto a polyvinylidene difluoride membrane (Millipore, Billerica, MA, United States). The membrane was blocked with 5% non-fat dry milk/0.1% Tween-20 in Tris-buffered saline for 2 h, at room temperature. Thereafter, the membrane was incubated with different primary antibodies, including rabbit anti-LC3B (1:1000 dilution, Sigma-Aldrich), rabbit anti-p62 (1:800 dilution, Sigma-Aldrich), rabbit anti-Parkin (1:500 dilution, Santa Cruz Biotechnology Inc., Santa Cruz, CA, United States), mouse anti-Cyt C (1:1000 dilution, Santa Cruz Biotechnology Inc., Santa Cruz, CA, United States), rabbit anti-Bax (1:500 dilution, Santa Cruz Biotechnology Inc., Santa Cruz, CA, United States) and mouse anti-Cox IV (1:500 dilution, Santa Cruz Biotechnology Inc., Santa Cruz, CA, United States), β-actin (1:3000 dilution, Santa Cruz Biotechnology Inc., Santa Cruz, CA, United States), rabbit anti-cleaved caspase-3 (1:1000 dilution, Cell Signaling Technology, Danvers), rabbit anti-cleaved caspase-9 (1:1000 dilution, Cell Signaling Technology), and rabbit anti-Apaf-1 (1:1000 dilution, Cell Signaling Technology). Subsequently, the membrane was treated with horseradish peroxidase-labeled secondary antibody for 2 h at room temperature. Immunoblots were probed using an enhanced ECL substrate (Pierce). The chemiluminescence level was recorded using an imaging system (Bio-Rad, Hercules, CA, United States). The results were normalized to a loading control β–actin or Cox-IV (mitochondrial control).

### Transmission Electronic Microscope

Mice were sacrificed and perfused transcardially with 4% paraformaldehyde and 0.5% glutaraldehyde in a 0.1 mol phosphate buffer,24 h post-SCIRI. The spinal cord tissue (T8-L4) was collected and coronal sections (100 μm) were cut by a vibratome, and postfixed with 4% glutaraldehyde in a 0.1 mmol cacodylate buffer (pH 7.4) for 1 h and incubated with 1% osmium tetroxide in a 0.1 mmol cacodylate buffer for 2 h. Spinal cord sections were dehydrated by an ascending series of ethanol and dry acetone and then embedded in Durcupan ACM Fluka (Sigma-Aldrich).Ultrathin sections (0.1 μm) were stained with uranyl acetate and lead citrate and subsequently examined with a JEOL JEM-1230 transmission electron microscope (JEOL, Tokyo, Japan).

### Immunohistochemistry Staining

Spinal cord tissues were collected 24 h after the SCIRI. The spinal cords (T8-L4) were fixed and sectioned into 15 μm slices using a Leica cryostat. Immunohistochemistry staining was performed as described previously (Han et al., 2016). Sections were blocked with 10% goat serum for 1 h at room temperature, followed by an incubation with primary antibodies overnight at 4°C and an incubation for 60 min at 25°C with secondary antibodies conjugated with the appropriate Alexa 594-conjugated secondary antibody (Molecular Probes, Eugene, OR, United States). The compound DAPI (Molecular Probes) was used to label cell nuclei. Primary antibodies were used in IHC as follows: rabbit anti- LC3 B (1:200 dilution, Sigma-Aldrich), mouse anti- Voltage-dependent anion channel (VDAC)1 (1:200 dilution, Millipore). Negative controls were performed without the primary antibody. The sections were examined under a fluorescence microscope (Eclipse 90i; Nikon, Tokyo, Japan).

### Terminal Deoxynucleotidyl Transferase dUTP Nick End Labeling (TUNEL) Assay

A TUNEL assay is the most commonly used technique for examining apoptosis via DNA fragmentation. TUNEL staining was performed using an In Situ Cell Death Detection kit (Roche, Shanghai, China) for spinal cords 24 h after the SCIRI, according to established protocols (Zhang et al., 2018). After TUNEL labeling, cell nuclei were labeled with DAPI, and examined under a fluorescence microscope. The number of TUNEL positive cells in each section was counted. The TUNEL-positive cells were defined as cells double labeled with TUNEL and DAPI. The quantity of TUNEL-positive cells of three sections from each mouse were respectively counted at high magnification and used for analysis.

### Statistical Analysis

The Mann-Whitney U test was used to compare the neurologic scores and cell numbers. A quantitative analyses of the optical density of the Western blots were analyzed by one-way ANOVA, followed by a Bonferroni *post hoc* test (GraphPad Software, San Diego, CA, United States). Parametric data were presented as mean ± SD. A *p*-value of less than 0.05 was considered statistically significant.

## Results

### Rapamycin Enhanced Mitophagy After SCIRI

To determine the characteristics of the neuronal mitophagy, we performed an IHC co-staining with antibodies against the LC3B (autophagy marker) and VDAC1(mitochondrial marker) in the injured spinal cord. We found that cells expressing LC3 were increased both in the rapamycin-treated mice and in the vehicle-treated mice, compared with the sham controls. The population of cells co-staining LC3B and VDAC1 in the rapamycin-treated mice was obviously higher than that in the vehicle-treated mice (Figure 1B). Higher magnification revealed the accumulation of LC3B-VDAC1 positive punctate dots in the neuron (Figure 1B). We also examined neurons in the gray matter of spinal cord mitophagy structures, corresponding to LC3B-VDAC1 co-staining puncta used with TEM. TEM results showed that the autolysosome filled with membranous whorls and an abnormal autophagic vesicle was distributed in neurons of the gray matter (Figures 1C-b). Furthermore, a mitophagy structure was clearly observed, and that the partially degraded mitochondria was surrounded by double membranes (Figure 1C-c).

To quantitatively analyze the mitophagy level, the mitochondrial fraction was isolated from the spinal cord tissue and LC3-II expression was determined in the mitochondrial fraction of the spinal cord using Western blotting. We found that rapamycin treatment could significantly increase the level of LC3-II in the ischemic spinal cord (*p* < 0.01, Figure 1D). Furthermore, we found that rapamycin treatment could significantly increase LC3-II expression in the mitochondrial fraction of the spinal cord (*p* < 0.01, Figure 1D). The data indicated that rapamycin treatment markedly enhanced mitophagy after the SCIRI.

### Rapamycin Enhanced Mitophagy via Promoting p62, Parkin Translocation to the Mitochondria

**FIGURE 2:**
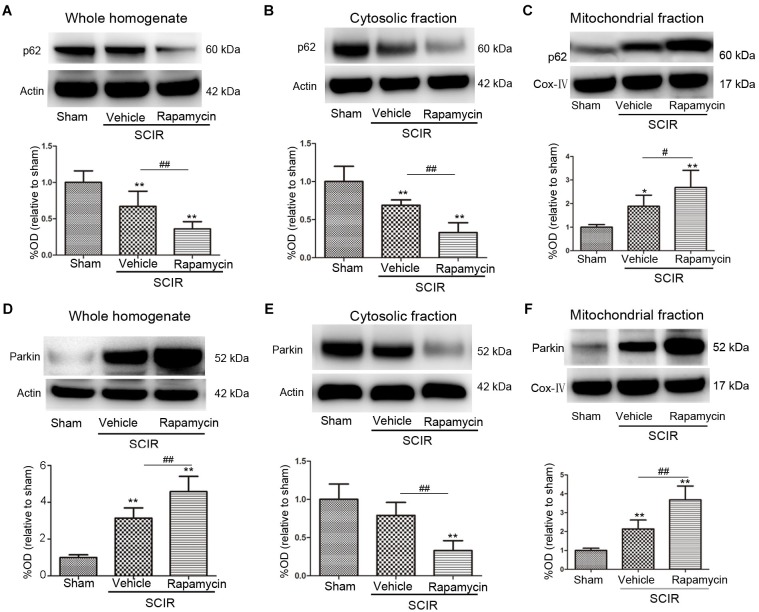
Rapamycin enhanced mitophagy via promoting p62, Parkin translocation to the mitochondria. **(A–C)** Representative western blots and quantitative graphs demonstrate the expression of p62 in the whole homogenates, in the cytosolic fractions and in the mitochondrial fractions at 24 h after SCIRI. Data were shown as mean ± SD, *n* = 6. ^∗^*p* < 0.05, ^∗∗^*p* < 0.01 vs; Sham, ^#^*p* < 0.05, ^##^*p* < 0.01. **(D-F)** Representative western blots and quantitative graphs demonstrate the expression of Parkin in the whole homogenates, in the cytosolic fractions and in the mitochondrial fractions at 24 h after SCIRI. Cox- IV is as inner control for mitochondria protein, β-actin is as inner control for tissue homogenate protein. Data were shown as mean ± SD, *n* = 6. ^∗^*p* < 0.05, ^∗∗^*p* < 0.01 vs. Sham; ^#^*p* < 0.05, ^##^*p* < 0.01.

To explore the mechanism of mitophagy activated by rapamycin, we also measured the mitochondrial p62 and Parkin after the SCIRI, which are key mediators for mitophagy (Ashrafi and Schwarz, 2013). The p62 as an adapter protein, can combine with damaged mitochondrial degraded by autophagy. Our results showed that rapamycin markedly promoted p62 accumulation in the mitochondrial fraction (*p* < 0.05, Figures 2A–C). Interestingly, ischemia-reperfusion induced the increase of the Parkin expression in the whole homogenate (*p* < 0.01, Figure 2D). Compared between the cytosolic and mitochondrial fraction, the results showed that rapamycin treatment caused a significant Parkin reduction in the cytosolic fraction and an increase in the mitochondrial fraction (*p* < 0.01, Figures 2E,F). These data suggested that rapamycin treatment significantly increased the Parkin expression in the mitochondria fraction after SCIRI. Overall, the data revealed that rapamycin treatment significantly enhanced mitophagy by increasing the translocation of p62 and Parkin to the damaged mitochondria.

### Rapamycin Inhibited the Release of Apoptosis-Related Proteins From Mitochondria

**FIGURE 3:**
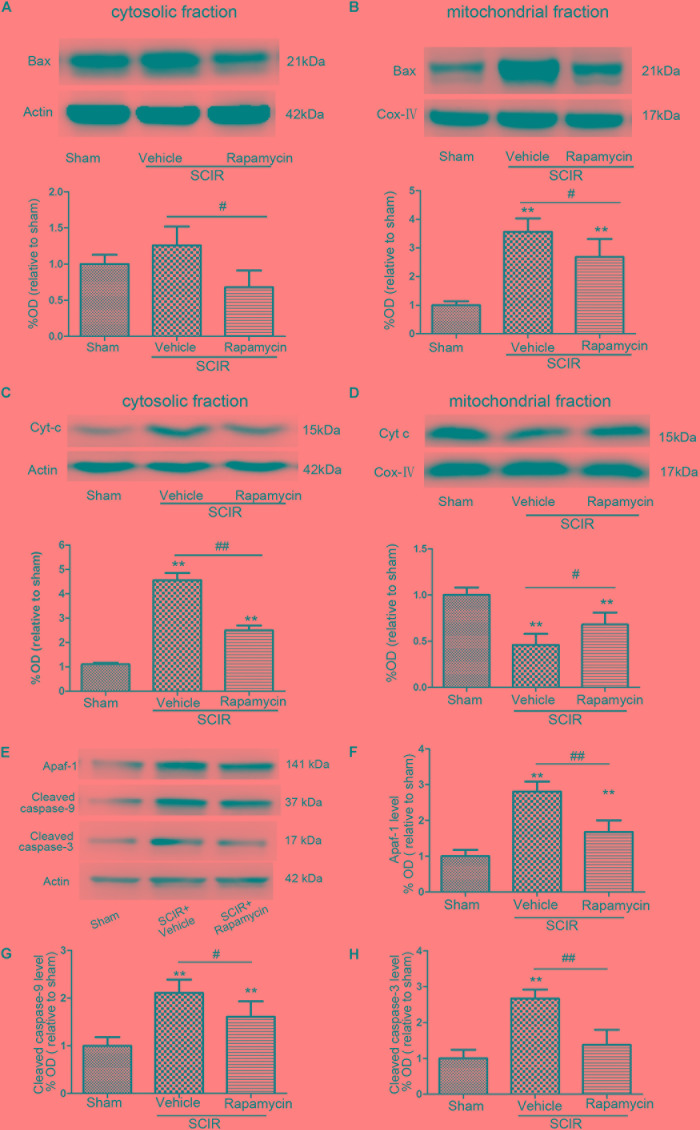
Rapamycin treatment following spinal cord ischemic injury reduced mitochondrial release of apoptosis related proteins. **(A,B)** Representative western blots and quantitative graphs demonstrate the expression of Bax in cytosolic and mitochondrial fractions at 24 h after SCIRI. Data are presented as the mean ± SD, *n* = 6. ^∗^*p* < 0.05, ^∗∗^*p* < 0.01 vs. Sham; ^#^*p* < 0.05, ^##^*p* < 0.01. **(C,D)** Representative western blots and quantitative graphs demonstrate the expression of Cyt-c in cytosolic and mitochondrial fractions at 24 h after SCIRI. Data are presented as the mean ± SD, *n* = 6. ^∗^*p* < 0.05, ^∗∗^*p* < 0.01 vs. Sham; ^#^*p* < 0.05, ^##^*p* < 0.01. **(E–H)** Representative western blots and quantitative graphs demonstrate the expression of mitochondrial apoptosis related proteins (Apaf-1, cleaved Caspase-3, cleaved Caspase-9) at 24 h after SCIR. Data are presented as the mean ± SD, n = 6. ^∗^*p* < 0.05, ^∗∗^*p* < 0.01 vs. Sham; ^#^*p* < 0.05, ^##^*p* < 0.01.

The mitochondria-dependant apoptosis is a well-accepted mechanism underlying neuronal death induced by an ischemia-reperfusion injury. The mitochondria transmit apoptotic signals through the release of cytochrome *c* to the cytoplasm during ischemia-reperfusion (Carloni et al., 2008). The release of cytochrome *c* correlates closely to the translocation of the proapoptotic proteins Bax to the mitochondria. In this present study, the results showed that SCIRI caused a significant Bax reduction in the cytosolic fraction and Bax increase in the mitochondrial fraction, indicating that SCIRI induces a robust Bax translocation from the cytosol to the mitochondria. But rapamycin treatment remarkably reduced Bax translocation after SCIRI (Figures 3A,B). Cytochrome *c*, an early marker of mitochondria-dependant apoptosis, was determined both in the cytosolic fraction and the mitochondrial fraction of the spinal cord. As shown in Figures 3C,D, cytochrome *c* expression significantly increased in the cytosolic fraction and decreased in the mitochondrial fraction after SCIRI. But rapamycin treatment significantly inhibited cytochrome *c* release from mitochondria, induced by the ischemia-reperfusion injury(Figure 3D), which shows that rapamycin treatment decreased the cytochrome *c* level in the cytosolic fraction and increased in the mitochondrial fraction. Moreover, western blotting results revealed that rapamycin treatment significantly decreased mitochondrial apoptosis related proteins (Apaf-1, Caspase-3, Caspase-9) released in SCIRI mice (*p* < 0.01 and *p* < 0.05, respectively, Figures 3E–H). Therefore, these data indicated that the activation of mitophagy by rapamycin in the reperfusion phase, attenuated mitochondria-dependent apoptosis in the ischemic spinal cord.

### Rapamycin Attenuated Apoptosis and Improved Locomotor Function Following Spinal Cord Ischemia

**FIGURE 4:**
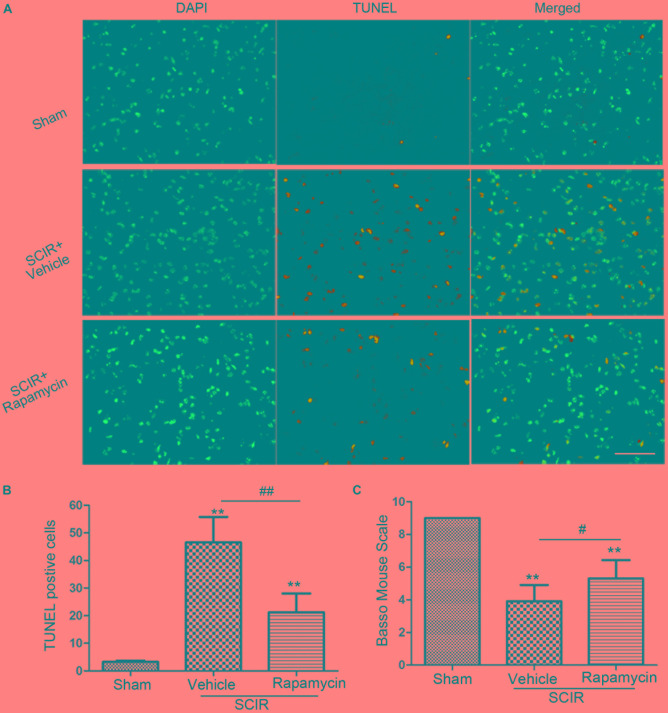
Rapamycin attenuated spinal cord ischemia-reperfusion induced apoptosis. **(A)**. These cells were stained with TUNEL and DAPI. The merged picture shows colocolization of apoptotic and nuclear markers. Scare bar = 50 μm. **(B)**.The bar graph shows rapamycin treatment rescued neurons from apoptosis at 24 h after SCIR. Data were shown as mean ± SD, *n* = 6. The numbers of apoptotic cells were counted in three sections of ischemic spinal cord. ^∗^*p* < 0.05, ^∗∗^*p* < 0.01 vs. Sham; ^#^*p* < 0.05, ^##^*p* < 0.01. **(C)** Bar graphs show the rapamycin-treated mice have significantly higher BMS scores than the vehicle-treated mice at 24 h after injury. Data were shown as mean ± SD, *n* = 16. ^∗^*p* < 0.05, ^∗∗^*p* < 0.01 vs. Sham; ^#^*p* < 0.05, ^##^*p* < 0.01.

Apoptosis underlies neuronal loss after SCIRI (Wu et al., 2014). We speculated that improved locomotor function by rapamycin treatment, may be attributed to the inhibition of cell apoptosis. To confirm this, a TUNEL assay was conducted to investigate the protective effect of rapamycin against apoptosis in SCIRI mice. We found marked TUNEL-positive cells in the spinal gray matter 24 h after SCIRI (Figure 4A). But rapamycin treatment significantly reduced TUNEL-positive cells after SCIRI (*p < 0.01*, Figure 4B). Additionally, BMS scores data, indicated the improvement of locomotor function, consistent with immunostaining results in the rapamycin-treated mice 24 h after SCIRI (*p* < 0.05, Figure 4C).

## Discussion

In the present study, we identified that autophagy and mitophagy were involved in the SCIRI process. Importantly, identified the protective roles of rapamycin on mitochondria after SCIRI: 1) rapamycin activated mitophagy by promoting p62 and Parkin translocation to the mitochondria; 2) rapamycin inhibited the release of apoptosis-related proteins from the mitochondria and attenuated mitochondria-dependent apoptosis in the ischemic spinal cord; and 3) rapamycin reduced neural tissue damage and improved locomotor function after SCIRI. The present study indicates that rapamycin treatment had a neuroprotective effect against SCIRI injury. These findings provide novel evidence that mitophagy activation can counteract apoptosis after a spinal ischemia-reperfusion injury.

Previous studies have demonstrated that the inhibition of the mTOR signaling, has a neuroprotective effect in the central nervous system (Carloni et al., 2008; Yin et al., 2012; Li et al., 2014). Some studies also demonstrate that rapamycin significantly reduces neuronal loss and cell death in the injured spinal cord (Sekiguchi et al., 2012; Wang et al., 2014). Simvastatin improves functional recovery through autophagy induction by inhibiting the mTOR signaling pathway after spinal cord injury in rats (Gao et al., 2015). Autophagy is involved in the pathophysiological process of SCIRI. Moreover, it was believed that autophagy plays opposing roles during the bimodal stage after SCIRI. Early activated autophagy alleviates spinal cord injury, but later activated autophagy excessively elevated autophagy aggravates I/R injury by inducing autophagic cell death (Fang et al., 2016). Concurrent with these findings, we found that enhancing autophagy by rapamycin treatment, immediately after reperfusion, exerts a protective role on neurons in a SCIR injury. Rapamycin treatment therefore restored neurological and motor function in SCIRI mice.

Given the benefits of autophagy activation via mTOR inhibition, the role of enhanced mitophagy of rapamycin during reperfusion, might imply a contribution to neuroprotection.

Mitophagy, the selective autophagy, is extremely crucial in maintaining mitochondrial homeostasis by removing damaged mitochondria and was implicated in the process of an ischemia-reperfusion injury (Zhang et al., 2013; Zuo et al., 2014; Yuan et al., 2015). Indeed, our results demonstrated that rapamycin could activate not only general autophagy but also mitophagy. In our study, we found marked LC3B-VDAC1 costaining puncta and a marked mitophagy structure under TEM in rapamycin-treated SCIRI mice. In addition, we found that rapamycin upregulated the LC3-II levels in a mitochondrial fraction, suggesting that mitophagosomes accumulated in spinal cord neurons. The key molecular mechanism for rapamycin-induced mitophagy activation is closely related to the p62 and Parkin pathway (Yuan et al., 2015; Tang et al., 2016). In this present study, we found that rapamycin significantly upregulated the p62 and Parkin translocation to the mitochondria after SCIRI injury. The phosphorylation of p62 has been proposed to initiate the binding of p62 to ubiquitinated proteins during autophagy and mitophagy (Ichimura et al., 2013). p62 contains a LC3 interacting domain and facilitates the recruitment of damaged mitochondria to the phagophore by binding to LC3-II (Ding et al., 2010; Geisler et al., 2010). Through polyubiquitinateion, Parkin can bind to the outer membrane of damaged mitochondria, followed by p62 binding to LC3-II, which can further result in damaged mitochondrial degradation via the autophagic machinery (Harper et al., 2018). A previous study has also demonstrated that mitophagy mediated by the Parkin protein, underlies the neuroprotection that occurrs in the process of cerebral ischemia reperfusion (Zhang et al., 2013). In addition to its role in mitophagy process, Parkin also stimulates mitochondrial biogenesis, presumably to replace damaged mitochondria with healthy and functional organelles (Shin et al., 2011). This effect of rapamycin could be examined in future studies.

Spinal cord ischemia reperfusion leads to neuronal death by inducing apoptosis (Wu et al., 2014; Foley et al., 2015). Importantly, mitochondria play a crucial role in regulating neuronal apoptosis (Zuo et al., 2014; Liu et al., 2015). Damaged mitochondria can release pro-apoptotic proteins to increase the activation of caspases and cell death (Adams and Cory, 2007; Anne Stetler et al., 2013). Removal of dysfunctional mitochondria is essential for cellular survival(Anne Stetler et al., 2013; Ashrafi and Schwarz, 2013). Therefore, mitophagy activation may subsequently attenuate apoptosis. It is supposed that the elimination of damaged mitochondria inhibits mitochondria-dependent apoptosis and subsequently promotes neuronal survival. In this study, results showed that rapamycin treatment significantly reduced Bax translocation to mitochondria and cytochrome *c* release from mitochondria in SCIRI mice. Additionally, rapamycin treatment significantly decreased the number of TUNEL-positive cells 24 h after SCIRI. Our results showed that rapamycin treatment resulted in neuroprotection and a significant functional recovery after SCIRI. We show here that these protective effects are linked to an increased mitophagic flux and inhibits mitochondria-dependent apoptosis. Our previous study reported that rapamycin could reduce brain injury after cerebral ischemia by promoting mitophagy (Li et al., 2014). The evidence in this study further demonstrated the central role of the mitochondria in neuronal survival after SCIRI. This work proves that the mitochondria is an important therapeutic target to protect against SCIRI. These findings increased our understanding of the relationship between mitophagy and neuroprotection after SCIRI.

## Conclusion

The present work showed that rapamycin administration during the acute phase of SCIRI could significantly reduce the neural tissue damage and locomotor impairment. Enhanced mitophagy by rapamycin could inhibit apoptosis and protect against SCIRI injury. Therefore, rapamycin treatment is a promising and effective therapeutic strategy for SCIRI injury.

## Author Contributions

JW and WS conceived and designed the research. QL, ZK, SG, MZ, XZ, and YZ performed the experiments. QL, G-YY, and JH analyzed the data and wrote the paper. QL and JW obtained the funding. All authors read and approved the final draft.

## Conflict of Interest Statement

The authors declare that the research was conducted in the absence of any commercial or financial relationships that could be construed as a potential conflict of interest.

## Abbreviations

ATP: adenosine triphosphate
BMS: Basso Mouse Scale
DAPI: 4′-6-diamidino-2-phenylindole
IHC: immunohistochemistry
LC3: microtubule-associated protein light chain 3
MCAO: middle cerebral artery occlusion
mTOR: mammalian target of rapamycin
ROS: reactive oxygen species
SCIRI: spinal cord ischemia-reperfusion injury
TEM: transmission electron microscopy
TUNEL: Terminal deoxynucleotidyl transferase dUTP nick end labeling
VDAC: Voltage-dependent anion channel
